# Supplementary material for: Neural activity tracking identity and confidence in social information
Source: eLife. 2023 Feb 10;12:e71315. doi: 10.7554/eLife.71315 (PMC9917428; doi:10.7554/eLife.71315)
Supplement: Supplementary file 1. — Relates to Figure 4 and Figure 4—figure supplement 1. Z-values and coordinates for exploratory fMRI-GLM1 for social and non-social conditions (family-wise error [FWE] cluster corrected with z > 2.3, p < 0.05). [file elife-71315-supp1.pdf]

Supplementary File 1, relates to Figure 4 and Figure 4 – figure supplement 1.

Contrast	Region	Peak Coordinates x/y/z (in mm MNI Space)	Z Value
**Social condition:** past judgment	dmPFC	2, 46, 34	-4.1
	Right pTPJ	48 -64 42	-4.4
	Inferior Parietal gyrus (left)	-46 -58 48	-3.8
	Precuneus	-2, -64, 38	-4.6
	Dorsolateral prefrontal cortex	-46 34 -12	-3.6
	Left STS	-64 -26 -6	-3.5
**Social condition:** current judgment	Striatum (right)	6 18 0	+3.6
	ACC gyrus	4 40 14	+3.6
	Insular (right)	4 12 0	+3.8
**Non-social:** current judgment	ACC sulcus	6 42 18	+4.4
	Insular (right)	42 22 0	+4.1
	OFC (bilateral, here left)	-38 18 -12	+4.2

Family-wise error cluster corrected, $z > 2.3$, $p < 0.05$
