# Supplementary material for: Neural activity tracking identity and confidence in social information
Source: eLife. 2023 Feb 10;12:e71315. doi: 10.7554/eLife.71315 (PMC9917428; doi:10.7554/eLife.71315)
Supplement: Transparent reporting form [file elife-71315-transrepform1.docx]

***eLife’s* transparent reporting form**

We encourage authors to provide detailed information *within their submission* to facilitate the interpretation and replication of experiments. Authors can upload supporting documentation to indicate the use of appropriate reporting guidelines for health-related research (see [EQUATOR Network](http://www.equator-network.org/%20)), life science research (see the [BioSharing Information Resource](https://biosharing.org/)), or the [ARRIVE guidelines](http://www.plosbiology.org/article/info:doi/10.1371/journal.pbio.1000412) for reporting work involving animal research. Where applicable, authors should refer to any relevant reporting standards documents in this form.

If you have any questions, please consult our Journal Policies and/or contact us: [editorial@elifesciences.org](mailto:editorial@elifesciences.org).

**Sample-size estimation**

- You should state whether an appropriate sample size was computed when the study was being designed
- You should state the statistical method of sample size computation and any required assumptions
- If no explicit power analysis was used, you should describe how you decided what sample (replicate) size (number) to use

Please outline where this information can be found within the submission (e.g., sections or figure legends), or explain why this information doesn’t apply to your submission:

This information can be found in the Methods, section: participants.

**Replicates**

- You should report how often each experiment was performed
- You should include a definition of biological versus technical replication
- The data obtained should be provided and sufficient information should be provided to indicate the number of independent biological and/or technical replicates
- If you encountered any outliers, you should describe how these were handled
- Criteria for exclusion/inclusion of data should be clearly stated
- High-throughput sequence data should be uploaded before submission, with a private link for reviewers provided (these are available from both GEO and ArrayExpress)

Please outline where this information can be found within the submission (e.g., sections or figure legends), or explain why this information doesn’t apply to your submission:

The experiment was performed once. We will provide the raw rata used for the analyses in the OSF repository. We will deposit unthresholded fMRI maps of all contrasts depicted in the manuscript on NeuroVault.

We will also make the code available; we have already made the full Bayesian modelling pipeline available (<https://osf.io/d5qzw/?view_only=037ea3b875914623a06999cef97ac57f>). Some of the relevant behavioural regressors were derived from this pipeline. Other regressors were used from the trial-by-trial raw confidence judgements. We will make the code available to replicate all results depicted in the main manuscript. We will also provide a README file inside the repository for details of its use (as we have done before, for example see: https://osf.io/d5qzw/?view_only=037ea3

b875914623a06999cef97ac57f). We have noted this information in ‘Data availability’ and ‘Code availability’.

We have excluded participants from the data set and the exclusion criteria are stated in the Methods (section: participants; section: RSA).

**Statistical reporting**

- Statistical analysis methods should be described and justified
- Raw data should be presented in figures whenever informative to do so (typically when N per group is less than 10)
- For each experiment, you should identify the statistical tests used, exact values of N, definitions of center, methods of multiple test correction, and dispersion and precision measures (e.g., mean, median, SD, SEM, confidence intervals; and, for the major substantive results, a measure of effect size (e.g., Pearson's r, Cohen's d)
- Report exact p-values wherever possible alongside the summary statistics and 95% confidence intervals. These should be reported for all key questions and not only when the p-value is less than 0.05.

Please outline where this information can be found within the submission (e.g., sections or figure legends), or explain why this information doesn’t apply to your submission:

We used a mixture of three statistical tests:

a) paired sample t-test to compare social and non-social conditions;

b) one sample t-test to test significance within a group against zero;

c) and a repeated-measures ANOVA when there are multiple levels of each variable.

For all reported statistical results, we added which statistical tests we used. For all statistical tests, we report their summary statistics, exact p-values and 95% confidence intervals.

All figures that show behavioural or neural results show the mean across participants within conditions, s.e.m. across participants and individual data points (Figure 2,3).

(For large datasets, or papers with a very large number of statistical tests, you may upload a single table file with tests, Ns, etc., with reference to sections in the manuscript.)

**Group allocation**

- Indicate how samples were allocated into experimental groups (in the case of clinical studies, please specify allocation to treatment method); if randomization was used, please also state if restricted randomization was applied
- Indicate if masking was used during group allocation, data collection and/or data analysis

Please outline where this information can be found within the submission (e.g., sections or figure legends), or explain why this information doesn’t apply to your submission:

We assigned the order of social and non-social conditions in a counterbalanced manner across participants (see Methods, section: participants).

There was no masking used during group allocation/ collection/ analysis as the experimenter had to instruct the participant.

**Additional data files (“source data”)**

- We encourage you to upload relevant additional data files, such as numerical data that are represented as a graph in a figure, or as a summary table
- Where provided, these should be in the most useful format, and they can be uploaded as “Source data” files linked to a main figure or table
- Include model definition files including the full list of parameters used
- Include code used for data analysis (e.g., R, MatLab)
- Avoid stating that data files are “available upon request”

Please indicate the figures or tables for which source data files have been provided:

We will prepare the code for the data analysis and make it as soon as possible available on OSF as described in ‘Data and Code availability’.

We have provided a ’Source Data’ file for all data presented in Figure2 and Figure3.

The specifications and code for the computational model (Bayesian model) are already uploaded, see previous explanations. However, we will combine everything (as described earlier) and make it available on OSF/NeuroVault.
